# Loss of HAI-2 in mice with decreased prostasin activity leads to an early-onset intestinal failure resembling congenital tufting enteropathy

**DOI:** 10.1371/journal.pone.0194660

**Published:** 2018-04-04

**Authors:** Roman Szabo, Thomas H. Bugge

**Affiliations:** Proteases and Tissue Remodeling Section, National Institute of Dental and Craniofacial Research, National Institutes of Health, Bethesda, MD, United States of America; University of Central Florida, UNITED STATES

## Abstract

Prostasin (CAP1/PRSS8) is a glycosylphosphatidylinositol (GPI)-anchored serine protease that is essential for epithelial development and overall survival in mice. Prostasin is regulated primarily by the transmembrane serine protease inhibitor, hepatocyte growth factor activator inhibitor (HAI)-2, and loss of HAI-2 function leads to early embryonic lethality in mice due to an unregulated prostasin activity. We have recently reported that critical *in vivo* functions of prostasin can be performed by proteolytically-inactive or zymogen-locked variants of the protease. Here we show that the zymogen form of prostasin does not bind to HAI-2 and, as a result, loss of HAI-2 does not affect prenatal development and survival of mice expressing only zymogen-locked variant of prostasin (Prss8 R44Q). Indeed, HAI-2-deficient mice homozygous for R44Q mutation (*Spint2*^*-/-*^*;Prss8*^*R44Q/R44Q*^) are born in the expected numbers and do not exhibit any obvious developmental abnormality at birth. However, postnatal growth in these mice is severely impaired and they all die within 4 to 7 days after birth due to a critical failure in the development of small and large intestines, characterized by a widespread villous atrophy, tufted villi, near-complete loss of mucin-producing goblet cells, loss of colonic crypt structure, and bleeding into the intestinal lumen. Intestines of *Spint2*^*-/-*^*;Prss8*^*R44Q/R44Q*^ mice showed altered expression of epithelial junctional proteins, including reduced levels of EpCAM, E-cadherin, occludin, claudin-1 and -7, as well as an increased level of claudin-4, indicating that the loss of HAI-2 compromises intestinal epithelial barrier function. Our data indicate that the loss of HAI-2 in *Prss8*^*R44Q/R44Q*^ mice leads to development of progressive intestinal failure that at both histological and molecular level bears a striking resemblance to human congenital tufting enteropathy, and may provide important clues for understanding and treating this debilitating human disease.

## Introduction

Prostasin (Channel-Activating Protease-1 (CAP-1/PRSS8) and matriptase (ST14/MT-SP1/epithin) are trypsin-like membrane-anchored serine proteases specifically expressed in most mouse and human epithelia [1, 2]. Studies using genetically-modified mouse strains suggest that the two proteases are part of a single proteolytic cascade and play a central role in epithelial development and homeostasis. In outbred mouse strains, loss of either prostasin or matriptase function during development leads to perinatal lethality due to failure to establish epidermal barrier function and subsequent fatal dehydration [3–7]. Furthermore, studies using tissue-specific knockout mice or rats carrying inactivating mutation in *Prss8* gene, encoding prostasin, revealed that matriptase and prostasin play crucial roles in epithelial development and function in a great variety of tissues, including placenta, skin, salivary gland, intestines, lungs, and thymus [8–13]. Furthermore, loss-of-function mutations in *ST14* gene, encoding matriptase, in human patients with autosomal recessive ichthyosis and hypotrichosis (ARIH)/Ichthyosis, Follicular Atrophoderma, and Hypotrichosis (IFAH) and in horses with Naked Foal Syndrome indicate that the function of matriptase-prostasin proteolytic pathway in epithelial development may be evolutionarily conserved across mammalian species [14–18].

The activity of the matriptase-prostasin pathway during development is controlled by two transmembrane serine protease inhibitors, hepatocyte growth factor activator inhibitor (HAI)-1 and HAI-2. In mice, HAI-1 is essential for placental development and overall embryonic as well as postnatal survival [19–21]. Loss of HAI-2 is associated with an early embryonic lethality on or before embryonic day (E) 8.5 in mice expressing normal levels of matriptase and prostasin, and with high frequency of neural tube defects, including exencephaly, spina bifida, and curly tail, as well as a mid-gestational embryonic lethality, due to a placental failure in matriptase-heterozygous mice [22–24]. All of these developmental defects in HAI-1- and HAI-2-deficient mice are rescued by simultaneous inactivation of either matriptase or prostasin, thus demonstrating a critical contribution of matriptase-prostasin proteolytic pathway to the developmental defects observed in these mice [22, 24]. Although HAI-1 and HAI-2 were shown to efficiently inhibit proteolytic activity of both matriptase and prostasin *in vitro* and in cell culture, epistatic analysis in mouse strains lacking either of the two proteases indicate that, at least during embryonic development, the two inhibitors play distinct roles, with HAI-1 acting predominantly as a direct inhibitor of matriptase, whereas HAI-2 regulates the activity of the pathway by targeting prostasin [25, 26]. Phenotypes of mice with genetic modification in matriptase, prostasin, HAI-1, and HAI-2 genes and their relative interactions are summarized in S1 Table [3–6, 11, 20–25, 27–32].

Mutations in the *SPINT2* gene, encoding HAI-2, have recently been described in a subset of patients with congenital tufting enteropathy (CTE) [33]. CTE presents in infants as a severe intestinal insufficiency associated with watery diarrhea, dehydration, and failure to thrive in the absence of parenteral feeding [34]. Histologically, CTE is characterized by epithelial dysplasia, varying degrees of villous atrophy and a compromised intestinal epithelial barrier. In over 70% of patients, the underlying mutation is in the *EPCAM* gene, encoding the epithelial cell adhesion molecule (EpCAM), a highly conserved cell surface glycoprotein involved in regulation of epithelial cell physiology [33, 35, 36]. In the absence of HAI-2, matriptase has been shown to cleave EpCAM in cultured intestinal epithelial cells, causing premature degradation of the tight junction protein claudin-7. Therefore, an increase in the activity of the matriptase-prostasin pathway, leading to an excessive cleavage of EpCAM protein and destabilization of tight junctions, has been proposed as the etiology of CTE in patients with *SPINT2* mutations [37].

We previously generated a knock-in mouse strain that carries a point mutation resulting in the substitution of arginine 44 in the activation cleavage site with glutamine (*Prss8*^*R44Q*^) [5, 31]. The intent was to generate a prostasin variant that is “locked” in the zymogen conformation by being resistant to activation site cleavage. Compatible with achieving this aim, R44Q prostasin in tissues from *Prss8*^*R44Q/R44Q*^ mice displayed a mobility in SDS/PAGE that was similar to the zymogen form of prostasin [5, 31]. Furthermore, the phenotype of *Prss8*^*R44Q/R44Q*^ mice was identical to a knock-in mouse strain that carries a point mutation resulting in the substitution of the catalytic serine 238 with alanine (*Prss8*^*S238A*^) [5, 31], indicating that R44Q prostasin does not attain full biological activity. Nevertheless, it cannot be excluded that R44Q prostasin in some tissues may undergo activation site cleavage by certain proteases with chymotryptic specificity [38]. Surprisingly, the phenotypes of *Prss8*^*R44Q/R44Q*^ or *Prss8*^*S238A/S238A*^ mice do not match those of prostasin null mice (*Prss8*^*-/-*^). Thus, both *Prss8*^*R44Q/R44Q*^ and *Prss8*^*S238A/S238A*^ mice are fully viable and only exhibit a mild defect in skin and hair development [5, 25, 31], indicating that critical prostasin biological functions are independent of its proteolytic activity.

In this study, we show that, unlike the wildtype and proteolytically-inactive prostasin, the zymogen-locked (R44Q) variant of prostasin is not an effective target for inhibition by HAI-2. As a result, loss of HAI-2 does not affect embryonic development and prenatal survival of *Prss8*^*R44Q/R44Q*^ mice. However, loss of HAI-2 leads to an inability to gain weight and death within 4 to 7 days after birth. *Spint2*^*-/-*^*;Prss8*^*R44Q/R44Q*^ pups present with distended colon and highly abnormal structure of epithelial compartments of both small and large intestine. Furthermore, the mice exhibit a progressive loss of EpCAM, E-cadherin, and claudin-7, indicating that the phenotype of Spint2^-/-^; Prss8^R44Q/R44Q^ mice recapitulates early events in the development of CTE in humans and provides an excellent model for study of the etiology and treatment of this debilitating disease.

## Materials and methods

### Mouse strains

All experiments were performed in an Association for Assessment and Accreditation of Laboratory Animal Care International-accredited vivarium following Standard Operating Procedures and were approved by the NIDCR Institutional Animal Care and Use Committee. HAI-2-deficient (*Spint2*^*-/-*^) and knockin mice expressing zymogen-locked prostasin (*Prss8*^*R44Q/R44Q*^) have been described in detail previously [24, 31]. All studies used mice of mixed 129S6/Sv;NIH BlackSwiss;FVB/NJ;C57Bl/6J genetic background and were littermate controlled. Ear or tail clips of newborn to two-week-old mice were subjected to genomic DNA extraction and genotyped by PCR as described elsewhere [3, 24, 31].

### Immunohistochemistry

Embryonic day (E) 18.5 to post-natal day (P) 4 old mice were euthanized, intestinal tissues extracted and immediately fixed in aqueous-buffered zinc formalin fixative (Z-Fix, Anatech Ltd., Battle Creek, MI) for 24 hours at room temperature and paraffin embedded (Histoserv Inc., Germantown, MD). 5 μm paraffin sections were stained for hematoxylin&eosin (H&E), alcian blue/PAS, and terminal deoxynucleotidyl transferase dUTP Nick-End Labeling (TUNEL) (all performed by Histoserv Inc.). Alternatively, the sections were immunostained after antigen retrieval by incubation for 20 min at 100°C in 0.01 M sodium citrate buffer, pH 6.0 essentially as described previously [25] (see S2 Table for information on antibodies). Three to five mice per each genotype and time point were analyzed. Number of proliferating and apoptotic cells was determined by manually counting Ki67-positive, and cleaved caspase-3- and TUNEL-positive cells, respectively, in five non-overlapping fields of small intestine in each sample.

### Protein extraction and Western blot analysis of mouse intestinal tissues

Small and large intestines were collected from E18.5, P2, and P4 mice, snap-frozen in liquid nitrogen, and stored at -80°C until further use. For Western blot analysis, the tissues were homogenized in buffer containing 2% SDS and 10% glycerol in 62.5 mM Tris/Cl pH 6.8. The lysates were cleared by centrifugation at 20,000 g for 10 min at 4°C to remove the tissue debris and the protein concentration in supernatant was determined by BCA assay (Pierce, Rockford, IL). 80 μg of total protein was loaded on 4–12% reducing SDS-PAGE and analyzed by Western blotting, incubating with primary antibody overnight at 4°C, followed by incubation with secondary antibody conjugated to alkaline phosphatase for 1.5 h at room temperature (see S2 Table for information on antibodies). Alkaline phosphatase activity was visualized using nitro-blue tetrazolium and 5-bromo-4-chloro-3'-indolylphosphate substrates (Sigma-Aldrich, St. Louis, MO). Data shown are representative of at least two independent Western blot experiments run on tissue lysates from three separate mice per each genotype and time point.

### Formation of prostasin inhibitory complexes *in vitro*

The preparation of wildtype, S238A, and R44Q mutated variants of human prostasin has been described in detail before [22, 39, 40]. Recombinant human HAI-1, HAI-2, and serpin E2/PN-1 were purchased from R&D Systems (Minneapolis, MN). To detect prostasin/HAI and prostasin/PN-1 inhibitory complexes, PI-PLC-released pro-prostasin variants were left untreated or first activated by incubation with 10 nM human recombinant matriptase serine protease domain (R&D Systems) for 20 minutes at 37°C. 100 ng of prostasin zymogen or matriptase-activated prostasin in 50 mM Tris/HCl, pH 8.0, 100 mM NaCl buffer was then incubated with 200 ng of human recombinant PN-1, HAI-1, or HAI-2 (all R&D Systems) for 30 min at room temperature. Reduced/non-boiled samples were analyzed by Western blotting as described above. Presented data are representative of two independent experiments.

### Analysis of HAI-2/prostasin interaction in mouse embryonic tissues

Protein extraction from the embryonic portion of mouse placenta was performed as described in detail in [22]. Combined lysates from three placentae of the same genotype, corresponding to 5 mg of total protein as determined by BCA assay (Pierce, Thermo Scientific, Rockford, IL), were diluted to 5ug/ul in 50 mM Tris/HCl, pH 8.0; 1% NP-40; 500 mM NaCl and pre-incubated with 50 μl GammaBind G Sepharose beads (GE Healthcare Bio-Sciences, Uppsala, Sweden) for 30 minutes at 4 ^o^C with gentle agitation. The samples were spun at 1,000 g for 1 min to remove the beads, and the supernatant was then incubated with 3 μg goat anti-mouse HAI-2 or goat anti-mouse prostasin antibody (both R&D Systems) and 50 ul of GammaBind G Sepharose beads for 3 hours at 4 ^o^C. The samples were spun at 1,000 g for 1 min, the supernatant was removed, and the beads were washed 3 times with 1 ml ice-cold 50 mM Tris/HCl, pH 8.0; 1% NP-40; 500 mM NaCl buffer. The beads were then mixed with 40 ul of 1x SDS loading buffer (Invitrogen, Carlsbad, CA) with 0.25 M β-mercaptoethanol, incubated for 5 min at 99 ^o^C, and cooled on ice for 2 minutes. Western blot analysis using mouse anti-human prostasin (1:250, BD Transduction Labs) or goat anti-mouse HAI-2 (1:500, R&D Systems) primary antibodies was performed as described above. Data shown are representative of two independent Western blot experiments run on tissue lysates from three separate placentae per each genotype. Western blot analysis of the starting material shown in S1 Fig was performed using 60 ug of tissue lysate.

### RNA preparation and quantitative RT-PCR

Small and large intestines collected postnatal day (P)2 mice were homogenized in Trizol reagent (Life Technologies, Grand Island, NY) and total RNA was extracted according to the manufacturer’s instructions. 1 ug of RNA was reverse transcribed with oligo dT primer using RETROscript kit (Invitrogen, Carlsbad, CA). Real-time PCR was conducted on 0.5 ul of cDNA template using iQ SYBR Green Supermix (Bio-Rad Laboratories, Hercules, CA) and 7500 Real-Time PCR System with 7500 Software v2.3 (Applied Biosystems, Foster City, CA). Primer sets used for cDNA amplification are listed in S3 Table. The expression of each gene was normalized to expression of ribosomal protein S15 with the ΔCt method. The assay was performed in triplicate. Results shown represent means and standard deviations from three independent samples per genotype.

### Enzymatic de-glycosylation assay

Wildtype recombinant human prostasin zymogen was prepared and activated by matriptase as described above. 100 ng of zymogen or of activated, double-chain prostasin was then subjected to removal of N- and O-linked carbohydrates using Enzymatic Protein Deglycosylation Kit (EDEGLY, Sigma-Aldrich, St. Louis, MO) according to manufacturer’s instructions. Resulting products were then analyzed by reducing SDS-PAGE and anti-prostasin Western blot analysis as indicated above.

### Statistical analysis

To evaluate the effect of HAI-2-deficiency on the embryonic survival of prostasin wildtype and prostasin zymogen-locked mice, chi-square analysis was performed on the observed versus the expected distribution of HAI-2 genotypes (*Spint2*^*+/+*^, *Spint2*^*+/-*^, and *Spint2*^*-/-*^) in mice carrying at least one wildtype allele (*Prss8*^*+/+*^ or *Prss8*^*R44Q/+*^, labeled as *Prss8*+) or homozygous for R44Q allele (*Prss8*^*R44Q/R44Q*^) of prostasin.

Initial body weight at birth (N ≥ 13 for each genotype) and the length of small and large intestines (N ≥ 5 for each genotype and time point), as well as the immunohistological, Western blot, and qPCR analysis were statistically evaluated using a two-sample Student’s t-test, two-tailed.

Survival of the *Spint2*^*−/−*^*;Prss8*^*R44Q/R44Q*^ and their HAI-2-expressing (*Spint2*^*+/+*^ or *Spint2*^*+/-*^, collectively labeled as *Spint2*+) and prostasin-expressing (*Prss8*^*+/+*^ or *Prss8*^*R44Q/+*^, collectively labeled as *Prss8*+) littermate control (i.e. *Spint2*^*+*^*;Prss8*^+^*)* mice after birth (N ≥ 13 for each genotype) was analyzed using Gehan-Breslow-Wilcoxon test (GraphPad Prism ver.7.03, GraphPap Software, Inc., La Jolla, CA).

Number of proliferating cells was determined by manually counting Ki67-positive cells in five non-overlapping fields of small intestine in each sample and the observed values were statistically evaluated using a two-sample Student’s t-test, two-tailed.

Western blot protein signal quantification was performed using ImageJ 1.46r software and statistically analyzed using a two-sample Student’s t-test, two-tailed.

## Results

### Binding of HAI-1 and HAI-2 to prostasin requires zymogen conversion but not proteolytic activity of the protease

**Fig 1:**
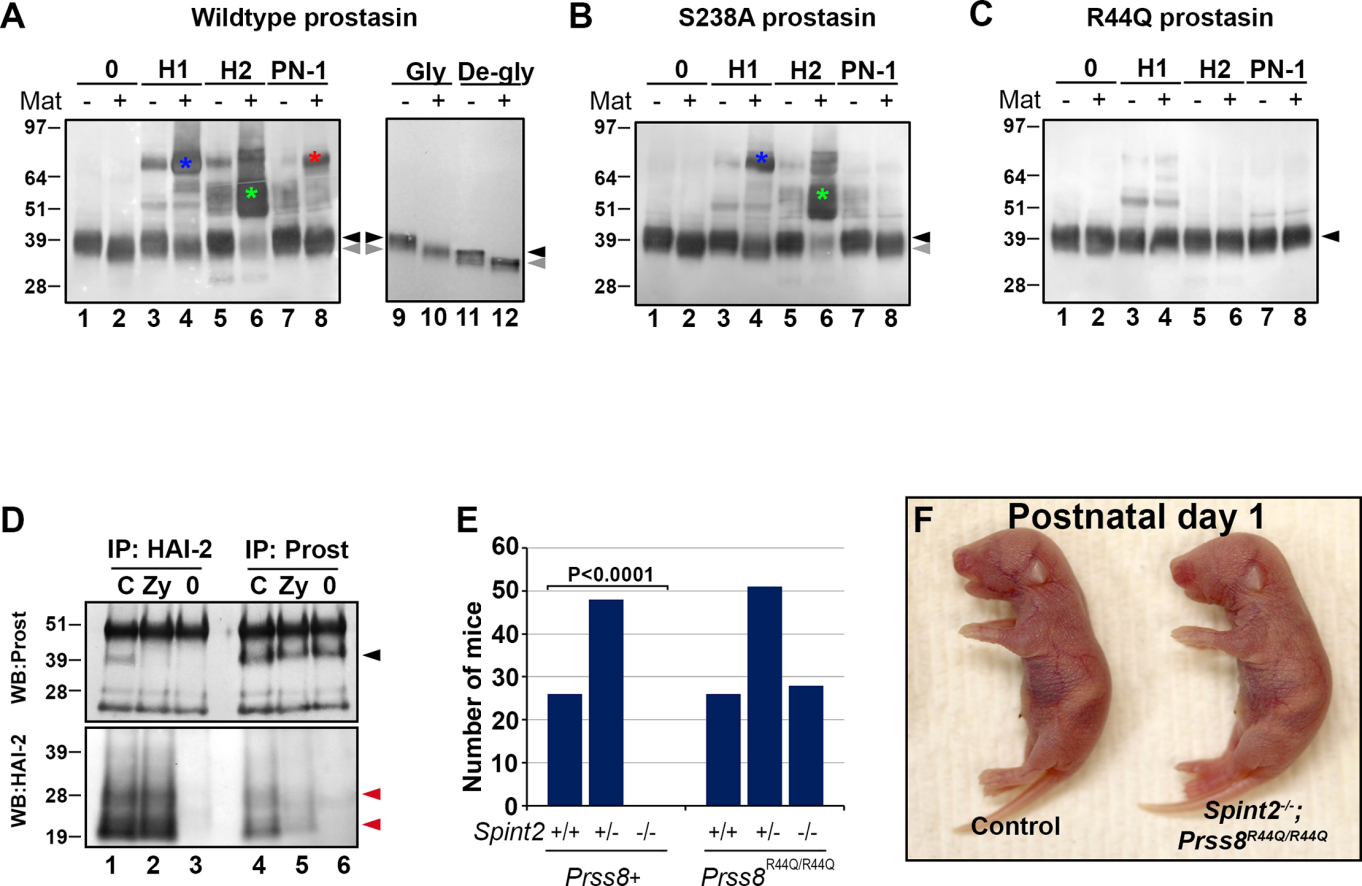
HAI-2 is dispensable for pre-natal development in *Prss8*^*R44Q/R44Q*^ mice. **(A-C).** Western blot detection of SDS-stable complexes between HAI-1 (H1), HAI-2 (H2), and protein nexin-1 (PN-1) and wildtype **(A)**, catalytically-inactive S238A **(B)**, and zymogen-locked R44Q **(C)** variants of prostasin after pre-incubation with (**A-C**, lanes 2, 4, 6, and 8) or without (**A-C**, lanes 1, 3, 5, and 7) recombinant human matriptase. HAI-1 and HAI-2 efficiently formed SDS-stable complexes with wildtype and catalytically-inactive prostasin after zymogen conversion (**A** and **B**, lanes 4 and 6), whereas PN-1 only formed complex with a wildtype prostasin (**A** and **B**, lane 8). No complexes were detected between the R44Q variant of prostasin and any of the three inhibitors (**C**, lanes 4, 6, and 8). Incubation with matriptase leads to a reduction in apparent molecular weight of prostasin both before (**A**, lanes 9 and 10) and after (**A**, lanes 11 and 12) de-glycosylation, indicating proteolytic processing of prostasin zymogen. Positions of prostasin zymogen (black arrowhead) and activated double-chain prostasin (grey arrowhead) are indicated on the right. Location of prostasin/HAI-1 (blue asterisk), prostasin/HAI-2 (green asterisk) and prostasin/PN-1 (red asterisk) are shown directly on the blot. Positions of protein molecular weight markers is shown on the left. **(D)**. Western blot detection of prostasin and HAI-2 after co-immunoprecipitation from E11.5 mouse placental tissues. Placental extracts from control (*Spint2*^*+/+*^*;Prss8*^*+/+*^, C, lanes 1 and 4), and HAI-2-expressing (*Spint2*^*+/+*^*;Prss8*^*R44Q/R44Q*^ (Zy, lanes 2 and 5) or HAI-2-deficient (*Spint2*^*-/-*^*; Prss8*^*R44Q/R44Q*^, 0, lanes 3 and 6) prostasin zymogen-locked embryos were incubated with anti-HAI-2 (lanes 1–3) or anti-prostasin (lanes 4–6) antibody, then analyzed by Western blot using anti-prostasin (black arrowhead, top panel) or anti-HAI-2 (red arrowheads, bottom panel) antibodies. The two proteins co-immunoprecipitated in mice expressing wildtype, but not R44Q prostasin. **(E)**. Distribution of HAI-2 genotypes among newborn mice from *Spint2*^*+/−*^; *Prss8*^*R44Q/+*^ breeding pairs. Loss of HAI-2 (*Spint2*^*-/-*^) leads to a complete embryonic lethality in mice expressing at least one wildtype allele (*Prss8*^*+/+*^ or *Prss8*^*R44Q/+*,^ collectively labeled as *Prss8+*) of prostasin (*Spint2*^*-/-*^;*Prss8+*, P<0.0001, χ^2^) but not zymogen-locked prostasin (*Spint2*^*-/-*^*;Prss8*^*R44Q/R44Q*^). **(F)**. Macroscopic appearance of newborn *Spint2*^*-/-*^*;Prss8*^*R44Q/R44Q*^ pups (right) and their wildtype littermate controls (*Spint2*^*+*^*;Prss8*^*+*^ left). No obvious developmental abnormalities associated with the loss of HAI-2 was noticed at birth.

We have previously reported that the developmental abnormalities and pre-natal lethality associated with embryonic loss of HAI-2 are averted by genetic inactivation of either matriptase or prostasin, thus identifying the matriptase-prostasin pathway as the only critical target of HAI-2 in pre-natal development [22, 24]. Specifically, as the phenotypes associated with prostasin deficiency are largely unaffected by loss of HAI-2 expression, we hypothesized that prostasin, rather than matriptase, serves as the primary target for HAI-2 inhibition [25]. Interestingly, most of prostasin’s physiological functions do not appear to require prostasin proteolytic activity or the proteolytic conversion of prostasin zymogen into its catalytically active double-chain form [5, 25, 31]. In order to further characterize the interaction of prostasin with HAI-2, we first tested the ability of different variants of prostasin to form inhibitory complexes with HAI-2, its closest homolog HAI-1, and a previously reported cognate serpin-type inhibitor of prostasin, protease nexin (PN)-1 [41, 42]. Thus, recombinant wildtype, S238A, and R44Q prostasin variants in their native zymogen form or converted into the double-chain forms by matriptase were incubated with the three inhibitors, followed by detection of inhibitory complexes by Western blot using prostasin antibody. As previously reported, wildtype double-chain (dc)-prostasin readily formed SDS-stable complexes with all three inhibitors (Fig 1A). Binding to HAI-2 appeared to be particularly effective, with virtually all of the detectable prostasin protein sequestered into the complex (Fig 1A, lane 6). Loss of proteolytic activity, as expected, prevented binding of S238A prostasin to the serpin-type inhibitor PN-1, but did not significantly affect binding to either of the HAI proteins (Fig 1B, lanes 4, 6, and 8). Small amounts of complexes were also detected with unactivated wildtype and the S238A prostasin (Fig 1A and 1B, lanes 3, and 5), most likely due to a low-level conversion of prostasin zymogen in the absence of matriptase, as evidenced by a presence of a prostasin species with an apparent molecular weight corresponding to activated dc-prostasin in prostasin zymogen sample after de-glycosylation (Fig 1A, lanes 11 and 12). This is consistent with a near-complete inability of R44Q prostasin variant that is unable to undergo proteolytic cleavage by matriptase (and referred to herein as zymogen-locked prostasin) to form inhibitory complexes with HAI-1, HAI-2, or PN-1 (Fig 1C).

HAI-2 is essential for inhibition of prostasin activity during mouse embryogenesis [22]. In order to test whether conversion into the double-chain form is indeed necessary for prostasin to become a target for inhibition by HAI-2 *in vivo*, we next performed a co-immunoprecipitation assay to analyze the interaction between the two proteins in embryonic tissues extracted from mice expressing wildtype (control) or zymogen-locked (R44Q) variants of prostasin (for analysis of starting material see S1 Fig). Consistent with its role as HAI-2 target, prostasin was readily detectable in placental extracts from control mice after immunoprecipitation with an anti-HAI-2 antibody (Fig 1D, top panel, lane 1). In contrast, no prostasin co-immunoprecipitated with HAI-2 in tissues that only express R44Q prostasin (Fig 1D, top panel, lane 2), despite expressing prostasin protein at levels comparable to their littermate controls (Fig 1D, top panel, compare lanes 4 and 5). Similarly, although placental tissues from mice expressing wildtype and R44Q prostasin express comparable levels of HAI-2 protein (Fig 1D, bottom panel, compare lanes 1 and 2), the amount of the inhibitor that was detected after immunoprecipitation with an anti-prostasin antibody was dramatically reduced in tissues expressing only R44Q prostasin (Fig 1D, bottom panel, compare lanes 4 and 5). Thus, our data indicate that zymogen-locked prostasin is unable to efficiently form stable complexes with HAI-2 *in vitro* or *in vivo* and is therefore not likely to be a target for HAI-2 inhibition.

### Loss of HAI-2 in mice expressing zymogen-locked prostasin does not affect prenatal development and survival

Mice expressing only the R44Q variant of prostasin have recently been described as fully viable, consistent with the conclusion that the prostasin zymogen can accomplish all of the essential developmental functions of prostasin [31]. However, if conversion to a double-chain form is indeed critical for the efficient binding of prostasin to HAI-2, and prostasin is the only relevant target for HAI-2 during pre-natal development (see above), our data suggest that, unexpectedly, HAI-2 may become dispensable for development in *Prss8*^*R44Q/R44Q*^ mice that only express zymogen-locked prostasin. To test this hypothesis, we interbred mice carrying HAI-2 null alleles (*Spint2*^*+/−*^) with mice homozygous for the *Prss8*^*R44Q*^ allele and analyzed offspring from the resulting *Spint2*^*+/−*^;*Prss8*^*R44Q/+*^ mice at birth.

Consistent with our previous reports, expression of wildtype prostasin protein led to a complete loss of prenatal viability in mice lacking HAI-2. Thus, no *Spint2*^*−/−*^ mice carrying two or one wildtype allele of prostasin (*Spint2*^*−/−*^*;Prss8*^*+/+*^ or *Spint2*^*−/−*^*;Prss8*^*R44Q/+*^) were observed at birth (Fig 1E, left panel, *P*<0.0001, χ^2^). However, HAI-2-deficient mice expressing only zymogen locked prostasin (*Spint2*^*−/−*^*;Prss8*^*R44Q/R44Q*^) did not exhibit any prenatal lethality, were born in the expected Mendelian ratio (Fig 1E, right panel, P = 0.92, χ^2^), and did not exhibit any gross developmental abnormality (Fig 1F). This apparent lack of requirement for HAI-2 to regulate prostasin activity in *Prss8*^*R44Q/R44Q*^ mice during embryogenesis is in support of the proposed hypothesis that the zymogen form of prostasin, while being biologically active, is not a developmental target for the inhibitor.

### HAI-2-deficient *Prss8* R44Q mice suffer from postnatal growth retardation and loss of viability due to intestinal failure

**Fig 2:**
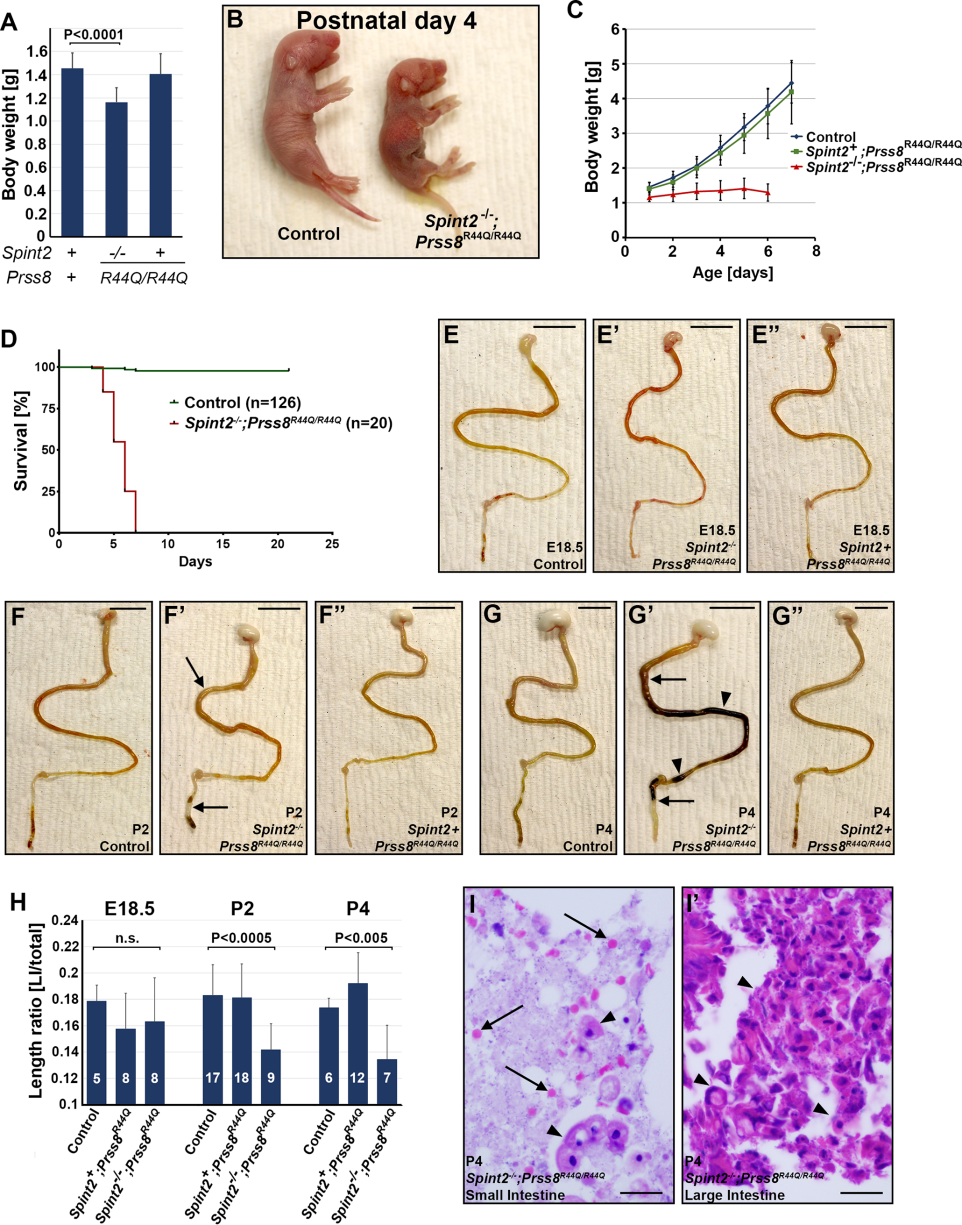
Loss of HAI-2 leads to defect in intestinal development and an early postnatal lethality. **(A)**. Body weight of control (*Spint2*^*+*^*;Prss8*^*+*^, n = 30), and prostasin zymogen-locked HAI-2-deficient (*Spint2*^*-/-*^*;Prss8*^*R44Q/R44Q*^, n = 13), and HAI-2-expressing (*Spint2*^*+*^*;Prss8*^*R44Q/R44Q*^, n = 47) mice at birth. HAI-2-deficient mice exhibit 20% reduction in the initial postnatal body weight (P<0.0001, Student’s t-test). **(B-D)**. Macroscopic appearance 4 days after birth **(B)**, postnatal weight gain **(C)**, and overall survival **(D)** of *Spint2*^*-/-*^*;Prss8*^*R44Q/R44Q*^ pups and their littermate controls. Loss of HAI-2 leads to postnatal growth retardation, inability to gain weight, and loss of viability 4–7 days after birth. **(E-G”)**. Macroscopic appearance of the gastrointestinal tract of control **(E-G)**, prostasin zymogen-locked HAI-2-deficient **(E’-G’)**, and HAI-2-expressing **(E”-G”)** mice on embryonic day (E)18.5 **(E-E”)**, postnatal day (P)2 **(F-F”)**, and postnatal day (P)4 **(G-G”)**. Intestines of HAI-2-deficient mice show signs of edema (**F’**, **G’**, black arrows), shortening of large intestine (**F’**, **G’**, left brackets) and intestinal bleeding (**G’**, black arrowhead) after birth. **(H)**. Relative length of large intestines (ratio of large intestine to total length of the intestines) in control, prostasin zymogen-locked HAI-2-expressing (*Spint2*^*+*^*;Prss8*^*R44Q/R44Q*^) and HAI-2-deficient (*Spint2*^*-/-*^*;Prss8*^*R44Q/R44Q*^) mice at E18.5, P2, and P4. The number of mice evaluated in each group and the p-values the observed differences between the controls and HAI-2-deficient mice are indicated. HAI-2-deficient mice exhibit significant shortening of the large intestine on P2 and P4. **(I, I’)**. H&E staining of the content of the lumen of the small **(I)** and large **(I’)** intestine of 4-days-old *Spint2*^*-/-*^*;Prss8*^*R44Q/R44Q*^ mice. Staining shows red blood cells (**I**, arrows) and high amount of cellular material (**I**, **I’**, arrowheads), indicating bleeding and excessive cell shedding into the lumen. Size bars: **(E-E”)** 8 mm; **(F-G”)** 10 mm; **(I, I’) 30** μm.

Although HAI-2-deficient *Prss8*^*R44Q/R44Q*^ mice were born in the expected numbers and were macroscopically otherwise unremarkable, they typically presented with about 20% reduction in body weight at birth compared to control and HAI-2-expressing *Prss8*^*R44Q/R44Q*^ littermates (Fig 2A, *P*<0.0001, Student’s t-test, 2 tailed). Furthermore, the postnatal growth of *Spint2*^*−/−*^*;Prss8*^*R44Q/R44Q*^ mice was severely impeded, despite presenting with a milk spot indicating ability to ingest food they consistently failed to gain any weight and all died 4 to 7 days after birth (Fig 2B–2D). Macroscopic inspection of the outward appearance of *Spint2*^*−/−*^*;Prss8*^*R44Q/R44Q*^ pups prior to their demise did not reveal any gross developmental abnormality and thus failed to provide an explanation for the growth retardation and early postnatal lethality (Fig 2B). Similarly, upon detailed macroscopic and histological examination, most major internal organs previously reported to express HAI-2, including brain, lungs, kidney and stomach, extracted from 4 days old *Spint2*^*−/−*^*;Prss8*^*R44Q/R44Q*^ mice appeared unremarkable. Loss of HAI-2 did, however, lead to a severe defect in the development of the lower gastrointestinal tract. The overall appearance of small and large intestines from *Spint2*^*−/−*^*;Prss8*^*R44Q/R44Q*^ mice before birth (embryonic day 18.5) was largely comparable with that of control or HAI-2-expressing *Prss8*^*R44Q/R44Q*^ littermate controls (Fig 2E–2E”). However, as early as on postnatal day 2, intestines from HAI-2-deficient mice appeared noticeably distended and the relative length of the large intestine was significantly reduced (Fig 2F–2F” and 2H). On postnatal day 4, intestines of most HAI-2-deficient mice were filled with dark material that contained high number of red blood cells, indicating bleeding into the lumen (Fig 2G–2G”, 2I and 2I’). No obvious abnormality was observed in the intestines from HAI-2-expressing *Prss8*^*R44Q/R44Q*^ mice at any point.

**Fig 3:**
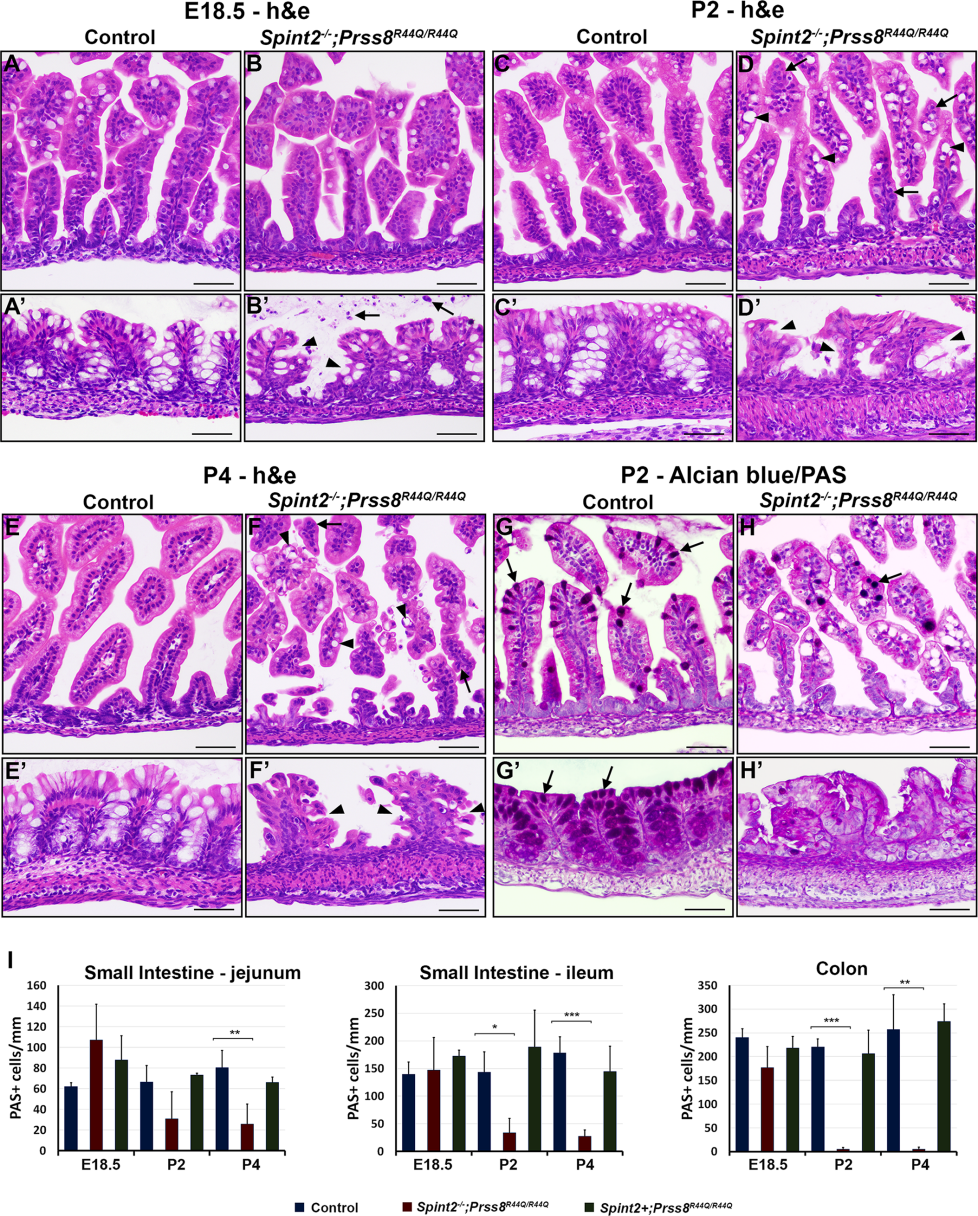
HAI-2 deficiency disrupts normal architecture of intestinal epithelium. **(A-F’).** H&E staining of small **(A-F)** and large **(A’-F’)** intestines from control and HAI-2-deficient (*Spint2*^*-/-*^*;Prss8*^*R44Q/R44Q*^) littermates at E18.5 **(A-B’)**, P2 **(C-D’)**, and P4 **(E-F’)**. After birth, loss of HAI-2 was associated with an increased dyslocalization of nuclei along the base of villous epithelium (**D**, **F**, arrows), accumulation of cells containing large vacuoles (**D**, **F**, arrowheads), and general villous atrophy of small intestine. In the large intestine, an increased shedding of cellular material into the lumen (**B’**, arrows) and a progressive loss of normal crypt structure (**B’**, **D’**, and **F’**, arrowheads) was observed in *Spint2*^*−/−*^*;Prss8*^*R44Q/R44Q*^ mice already as early as on E18.5. **(G-H’)**. Representative image of Alcian blue/PAS staining of small **(G, H)** and large **(G’, H’)** intestines from control **(G, G’)** or *Spint2*^*−/−*^*;Prss8*^*R44Q/R44Q*^ **(H, H’)** mice 2 days after birth (P2). HAI-2-deficiency is associated with a substantial decrease and a near complete loss of mucin-producing goblet cells (**G-H’**, arrows) in small and large intestine, respectively. **(I)**. Quantification of Alcian blue/PAS staining-positive cells in proximal (jejunum, left panel) and distal (ileum, middle panel) small intestine, and colon (right panel) at E18.5, P2, and P4. *Spint2*^*−/−*^*;Prss8*^*R44Q/R44Q*^ mice present with a substantially decreased number of PAS-positive cells in both small and large intestines after birth. Graphs show mean and standard deviation based on at least three animals per each genotype and time point. P values: * <0.05, **<0.01, ***<0.001. Scale bars: **(A-F’)** 75 μm, **(G-H’)** 50 μm.

Histological analysis of intestinal tissues from *Spint2*^*−/−*^*;Prss8*^*R44Q/R44Q*^ mice revealed no obvious abnormalities in the villous structure of small intestines at embryonic day 18.5 (Fig 3A and 3B). However, already at this time these mice presented with general disorganization of intestinal epithelium within crypts of the large intestine, associated with an increased shedding of cellular material into the lumen, decreased number of mucin-producing goblet cells, and lack of well-organized crypt structure present in HAI-2-expressing littermate control mice (Fig 3A’and 3B’). Analysis of intestinal tissues after birth revealed progressive changes in epithelia of both small and large intestine. On postnatal day 2, the small intestine showed signs of villous atrophy, increased dyslocalization of nuclei within the epithelial layer indicating loss of epithelial cell polarity, along with a substantial number of epithelial cells containing very large vacuoles and (Fig 3C and 3D). In the large intestine, this was associated with a disorganization of surface epitheliumand overall loss of crypt structure (Fig 3C’ and 3D’). The severity of the phenotypes increased over time, with widespread villous atrophy, abnormal invaginations between the enterocytes, tufted villi, enterocyte crowding, and essential loss of normal tissue architecture of both small and large intestines all manifesting by postnatal day 4 (Fig 3E–3F’). Abnormal differentiation of intestinal epithelium also manifested by, respectively, a significant reduction and a near complete absence of mucin-producing goblet cells in small and large intestines at P2 and P4 (Fig 3G–3I). Intestines from HAI-2-expressing *Prss8*^*R44Q/R44Q*^ mice did not exhibit any obvious histological abnormality at any point.

**Fig 4:**
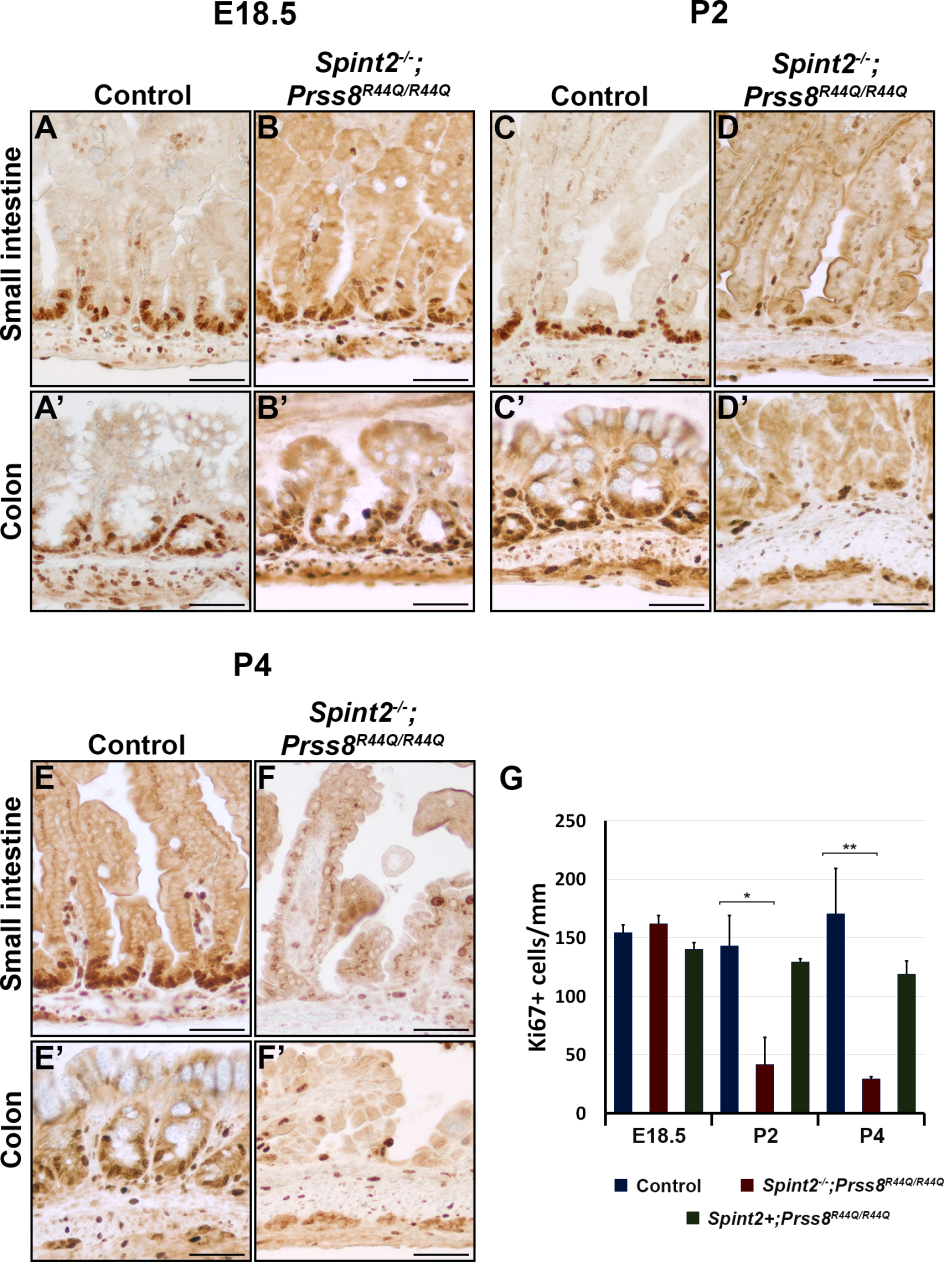
HAI-2 deficiency leads to decreased proliferation of intestinal epithelium. **(A-F’).** Anti-Ki67 immunostaining of E18.5 **(A-B’)**, P2 **(C-D’)**, and P4 **(E-F’)** tissues of small **(A-F)** and large **(A’-F’)** intestines from control and *Spint2*^*−/−*^*;Prss8*^*R44Q/R44Q*^ littermates. **(G)**. Quantitative analysis of proliferation in control and HAI-2-deficient intestinal tissues. Proliferation rate is determined as number of Ki67-positive epithelial cells (**A-F’**, examples with arrows) per mm of the length of the tissue. Loss of HAI-2 leads to, respectively, a 70% and an 80% reduction in proliferation within intestinal epithelium on P2 and P4. P values: * <0.05; ** <0.01. Size bars: **(A-F’)** 40 μm.

Immunohistochemical analysis of the expression of the cell proliferation marker Ki67 showed that loss of HAI-2 did not affect epithelial cell proliferation during pre-natal stages of development, as documented by a comparable number of Ki67-positive epithelial cells in control and *Spint2*^*−/−*^*;Prss8*^*R44Q/R44Q*^ mice at E18.5 (Fig 4A–4B’ and 4G). However, analysis of the postnatal tissues revealed a strong reduction in the proliferation rates of intestinal epithelial cells in *Spint2*^*−/−*^*;Prss8*^*R44Q/R44Q*^ mice after birth, with approximately 70 and 80% decrease in the number of Ki67-positive epithelial cells in both small and large intestines at postnatal day 2 and 4, respectively (Fig 4C–4G). On the other hand, despite widespread villous atrophy, substantial shedding of epithelial cells, and a general disorganization of intestinal epithelium, no apoptotic cells were detected by TUNEL or anti-p-caspase-3 stain in the intestinal tissues from two- or four-days-old *Spint2*^*−/−*^*;Prss8*^*R44Q/R44Q*^ mice, as well as from their control (*Spint2*^*+*^*;Prss8*^*+*^) and HAI-2-expressing prostasin zymogen-locked (*Spint2*^*+*^*;Prss8*^*R44Q/R44Q*^) littermates, suggesting that epithelial demise in these animals is not associated with the activation of an apoptotic program. No histological abnormalities were detected in intestines from HAI-2-expressing *Prss8*^*R44Q/R44Q*^ mice at any stage, suggesting that the observed changes result from the loss of HAI-2 expression rather than from the inability of R44Q prostasin to be converted into its double-chain form. Taken together, our histopathological analysis indicates that loss of HAI-2 in *Spint2*^*−/−*^*;Prss8*^*R44Q/R44Q*^ mice leads to severe defects in intestinal epithelial integrity consistent with those observed in CTE patients.

### Loss of HAI-2 function leads to altered expression of epithelial junctional proteins

**Fig 5:**
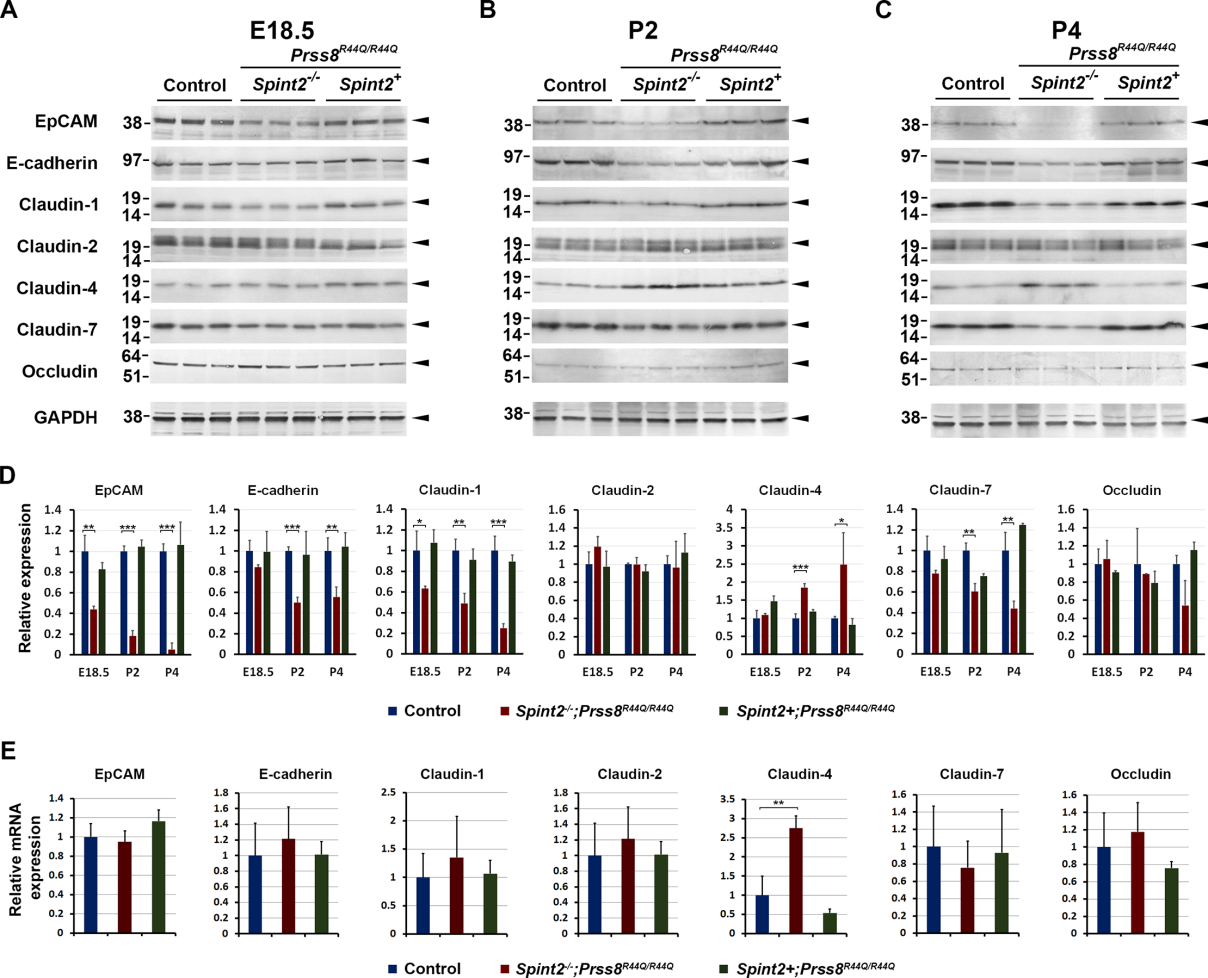
Loss of HAI-2 affects expression of intestinal epithelial tight and adherens junction proteins. **(A-C).** Western blot analysis of the expression of epithelial junctional proteins, from top to bottom, EpCAM, E-cadherin, claudin -1, -2, -4, and -7, and occludin in intestines from control (*Spint2*^*+*^*;Prss8*^+^, lanes 1–3), HAI-2-deficient (*Spint2*^*−/−*^*;Prss8*^*R44Q/R44Q*^, lanes 4–6), and HAI-2-expressing (*Spint2*^*−/−*^*;Prss8*^*R44Q/R44Q*^, lanes 7–9) prostasin zymogen-locked mice at E18.5 (**A)**, P2 **(B)**, and P4 **(C)**. Positions of the protein bands is indicated on the far right. Positions of molecular weight markers are indicated on the left. **(D)**. Quantification of the Western blot analysis shown in **A-C**. Expression in control (*Spint2*^*+*^*;Prss8*^*+*^) tissues shown in blue, HAI-2-deficient (*Spint2*^*−/−*^*;Prss8*^*R44Q/R44Q*^) in red, and HAI-2 expressing zymogen-locked (*Spint2*^*+*^*;Prss8*^*R44Q/R44Q*^) in green bars. *Spint2*^*−/−*^*;Prss8*^*R44Q/R44Q*^ mice presented with a significantly decreased expression of EpCAM and claudin-1 as early as on E18.5, followed by a decreased expression of E-cadherin and claudin-7, and an increased expression of claudin-4 on postnatal days 2 and 4. Changes in occludin expression did not reach statistical significance, and no changes were detected in the expression of claudin-2. Data are representative of at least two independent experiments. (E). Quantification of mRNA expression of junctional proteins in intestinal tissues from P2 control (blue), HAI-2-deficient (*Spint2*^*−/−*^*;Prss8*^*R44Q/R44Q*^, red), and HAI-2 expressing zymogen-locked (*Spint2+;Prss8*^*R44Q/R44Q*^, green) mice. Only the gene encoding claudin-4 was differentially expressed, showing significant up-regulation in HAI-2-deficient tissues. **D, E:** P values: * <0.05; ** <0.01; *** <0.001.

Improper intestinal barrier function has been implicated in the etiology of CTE [43]. Furthermore, unregulated matriptase activity leading to increased turnover of the epithelial junctional proteins EpCAM and claudin-7 has recently been implicated in the etiology of intestinal failure in a subset of congenital tufting enteropathy (CTE) patients carrying mutation in the *SPINT2* gene encoding HAI-2 [37]. As the developmental defects observed in HAI-2-deficient mice appear to closely mimic clinical features of CTE, we next analyzed expression of main components of epithelial tight and adherens junctions in intestines from *Spint2*^*−/−*^*;Prss8*^*R44Q/R44Q*^ mice and their healthy littermate controls at different developmental time points. At E18.5, before the mice are born and begin to ingest food, expression of most epithelial junctional proteins, including claudin-2, 4, 6, and 7, E-cadherin, and occludin appeared to be unaffected in the intestines from *Spint2*^*−/−*^*;Prss8*^*R44Q/R44Q*^ mice (Fig 5A). Loss of HAI-2 did, however, lead to a 56% and 37% reduction in the expression of EpCAM and claudin-1, respectively (Fig 5A and 5D). The levels of both proteins continued to decline after birth, with only 18% and 5% of EpCAM and 49% and 25% of claudin-1 protein detected at postnatal day 2 and 4, respectively, compared to HAI-2-expressing littermate controls (Fig 5B–5D). This was followed by a reduced expression of E-cadherin (50% on P2, 55% on P4), claudin-7 (60% on P2, 44% on P4) and occludin (unchanged on P2, 54% on P4) (Fig 5C and 5D). On the other hand, expression of claudin-2 was not affected during the progression of the disease, whereas the expression of claudin-4 gradually increased in HAI-2-deficient intestines compared to *Spint2*^*+*^*;Prss8*^*+*^ littermate controls (185% on P2, 248% on P4) (Fig 5A–5D). Further analysis of the gene expression showed no significant changes in mRNA levels in P2 intestinal tissues for any of the proteins down-regulated in the absence of HAI-2 (Fig 5E). Interestingly, however, mRNA for the sole junctional protein found upregulated in *Spint2*^*−/−*^*;Prss8*^*R44Q/R44Q*^ tissues, claudin-4, was increased 2.75-fold, compared to *Spint2*^*+*^*;Prss8*^*+*^ littermate controls (Fig 5E).

These data suggest that the loss of HAI-2 leads to a specific sequence of molecular events that are expected to affect integrity of epithelial cell-cell junctions and intestinal barrier function.

## Discussion

Recent genome editing studies in mice unexpectedly proposed that key developmental and postnatal functions of the membrane-anchored serine protease, prostasin, can be executed by prostasin variants that either are locked in the one-chain zymogen conformation or can undergo conversion to the active two-chain form, but are rendered catalytically-inactive by mutation of the active site serine residue [5, 31]. The current study, when combined with these and other previous studies [22, 24], supports the notion that the one-chain prostasin zymogen is refractory to HAI-2 regulation, while the two-chain prostasin is susceptible to HAI-2 regulation and is an essential developmental inhibitory target for the protease inhibitor. Although rendering prostasin zymogen-locked allowed HAI-2-deficient embryos to successfully pass previously established HAI-2-dependent early and mid-term developmental milestones, these mice all developed a congenital tufting enteropathy (CTE)-like phenotype leading to an inability to gain weight after birth and an early postnatal lethality. Furthermore, we found that reduction of EpCAM protein expression was an early event in the HAI-2-deficient intestine, and that this was followed by a progressive loss of claudin-7, claudin-1 and E-cadherin, but an increase in gene and protein expression of claudin-4. Given the essential role of junctional proteins in the development and maintenance of epithelial function [44–46], it is reasonable to propose that these molecular changes are, at least in part, responsible for the intestinal demise observed in *Spint2*^*−/−*^*;Prss8*^*R44Q/R44Q*^ mice.

Our analysis, thus, lends critical *in vivo* support to the central hypothesis generated from a recent cell-based study addressing the molecular consequences of HAI-2-insufficiency on intestinal epithelium. This study proposed EpCAM as a critical pathogenic substrate for matriptase during conditions of HAI-2 insufficiency, and linked the demise of epithelial cell-cell junctions in HAI-2-silenced intestinal cell monolayers directly site-specific cleavage of EpCAM by matriptase, which triggered endocytosis and lysosomal degradation of the cell adhesion molecule. This, in turn, caused destabilization of claudin-7, a key intestinal tight junction protein [37].

While our study does not directly address the identity of the protease or proteases responsible for the intestinal abnormalities observed in *Spint2*^*−/−*^*;Prss8*^*R44Q/R44Q*^ mice, it is reasonable to assume that, again, matriptase activity may play an important role. In addition to prostasin, matriptase has previously been identified as a very efficient target for HAI-2 inhibition *in vitro* [47]. As mentioned above, early loss of EpCAM protein, followed by a decrease in claudin-7 expression in *Spint2*^*−/−*^*;Prss8*^*R44Q/R44Q*^ mice mimic closely molecular changes induced by unregulated matriptase activity in HAI-2-silenced intestinal cell monolayers [37]. Furthermore, as HAI-2 does not appear to efficiently target prostasin in *Prss8*^*R44Q/R44Q*^ tissues, we do not expect that the loss of the inhibitor would directly trigger critical mis-regulation of prostasin activity. Analysis of the contribution of matriptase to the development of CTE-like phenotype in HAI-2-deficient mice is therefore of paramount importance and should be addressed in future studies.

Previous studies have revealed that insufficient intestinal matriptase proteolytic activity, like excessive activity implied for CTE patients, results in an increased paracellular permeability of intestinal epithelium followed by an epithelial demise [8, 28]. The superficially similar effects of blunted and excessive matriptase activity on intestinal epithelium and intestinal function raises the obvious possibility of a common etiology; that is the perturbation of EpCAM-dependent tight junction formation. In its most attractive scenario, matriptase would function as a processing protein in intestinal epithelium that converts EpCAM to its biologically active tight junction-inducing form, with HAI-2 serving to prevent excessive or aberrant proteolysis of EpCAM. Although this hypothesis is attractive and warrants further investigation, some observations indicate that this scenario may be too simplistic: First, we have failed to detect a change in EpCAM protein expression in mice with intestinal ablation of matriptase or prostasin (S1 Fig). Second, expression of claudin-2, a key regulatory target for matriptase in the normal intestine that accumulates under conditions of matriptase deficiency [28], was unaffected by the loss of HAI-2. Altogether, these findings indicate that the effects of the matriptase-prostasin system on epithelial cell-cell adhesion are complex and dynamic, and that, importantly, the target substrates for the system may differ under normal physiological conditions, and under conditions where this proteolytic system is dysregulated, such as complete or partial deficiency for HAI-2.

HAI-2 deficiency induced the loss of intestinal EpCAM already at late stages of embryonic development, showing that no environmental trigger, such as microbial colonization or food exposure, is required for initiation of the chain of molecular events leading to CTE. This suggests that strategies aimed at reshaping the microbiome to control intestinal permeability in individuals with CTE caused by HAI-2 deficiency are likely to be nonproductive. Rather, efforts should be focused on controlling the aberrant activity of the matriptase-prostasin axis. An obvious and attractive strategy would entail the oral administration of non-absorbable small molecule trypsin-like serine protease inhibitors with high activity towards matriptase and/or prostasin, and, correspondingly, a low activity towards trypsin and chymotrypsin. This approach may prove particularly useful for treatment of human patients, as matriptase deficiency in humans, unlike other species examined, has not been reported to be associated with overt gastrointestinal abnormalities [16–18].

In conclusion, we have shown that mice carrying a zymogen-locked version of prostasin are permissive for development in the absence of HAI-2, but that these HAI-2-deficient mice develop a CTE-like syndrome, possibly initiated by excessive proteolytic cleavage of EpCAM. Furthermore, our study provides an excellent animal model for the development of treatment strategies for this debilitating congenital disease.

## Supporting information

S1 Fig Western blot analysis of mouse placental and intestinal tissue lysates. **(A).** Western blot detection of prostasin (upper panel) and HAI-2 (lower panel) in placental tissue lysates (Start, lanes 1–3) and eluates from GammaBind g Sepharose beads after pre-incubation (Beads, lanes 4–6) from control (*Spint2*^*+/+*^*;Prss8*^*+/+*^, C, lanes 1 and 4), and HAI-2-expressing (*Spint2*^*+/+*^*;Prss8*^*R44Q/R44Q*^ (Zy, lanes 2 and 5) or HAI-2-deficient (*Spint2*^*-/-*^*; Prss8*^*R44Q/R44Q*^, 0, lanes 3 and 6) prostasin zymogen-locked embryos used for immunoprecipitation assay shown in Fig 1D. Positions of protein molecular weight markers are shown on the left. Expected size of prostasin and HAI-2 signal is indicated by black arrowhead (top panel) and red arrowheads (bottom panel), respectively. Low concentration and diffuse signal (compare to Fig 1D) prevents clear identification of HAI-2 in the starting material. Neither prostasin nor HAI-2 appear to non-specifically bind sepharose beads. (B). Western blot analysis of EpCAM expression in control (lanes 1 and 2), matriptase-deficient (Villin-Cre^+^;St14^fl/-^, lanes 3 and 4), prostasin-deficient (Villin-Cre^+^;Prss8^fl/-^, lanes 5 and 6), and matriptase and prostasin double-deficient (Villin-Cre^+^;St14^fl/-^; Prss8^fl/-^, lanes 7 and 8) P2 intestines. No obvious changes in the expression level or proteolytic processing of EpCAM protein have been noticed in any of the tissues. Positions of protein molecular weight markers are shown on the left.
(TIF)

S1 Table Spontaneous phenotypes observed in mice with genetic modification in genes encoding matriptase, prostasin, HAI-1 and HAI-2 and their interactions. (DOCX)

S2 Table List of antibodies used in the study. (DOCX)

S3 Table Sequences of PCR primers used for reverse transcription quantitative PCR. (DOCX)

## References

[1] Miller GS, List K. The matriptase-prostasin proteolytic cascade in epithelial development and pathology. Cell and tissue research. 2013;351(2):245–53. doi: 10.1007/s00441-012-1348-1 . 22350849 10.1007/s00441-012-1348-1

[2] List K, Hobson JP, Molinolo A, Bugge TH. Co-localization of the channel activating protease prostasin/(CAP1/PRSS8) with its candidate activator, matriptase. J Cell Physiol. 2007;213(1):237–45. doi: 10.1002/jcp.21115 . 17471493 10.1002/jcp.21115

[3] List K, Haudenschild CC, Szabo R, Chen W, Wahl SM, Swaim W, et al Matriptase/MT-SP1 is required for postnatal survival, epidermal barrier function, hair follicle development, and thymic homeostasis. Oncogene. 2002;21(23):3765–79. doi: 10.1038/sj.onc.1205502 12032844 10.1038/sj.onc.1205502

[4] Leyvraz C, Charles RP, Rubera I, Guitard M, Rotman S, Breiden B, et al The epidermal barrier function is dependent on the serine protease CAP1/Prss8. J Cell Biol. 2005;170(3):487–96. doi: 10.1083/jcb.200501038 . 16061697 10.1083/jcb.200501038 PMC2171460

[5] Peters DE, Szabo R, Friis S, Shylo NA, Uzzun Sales K, Holmbeck K, et al The membrane-anchored serine protease prostasin (CAP1/PRSS8) supports epidermal development and postnatal homeostasis independent of its enzymatic activity. J Biol Chem. 2014;289(21):14740–9. Epub 2014/04/08. doi: 10.1074/jbc.M113.541318 ; PubMed Central PMCID: PMCPMC4031529. 24706745 10.1074/jbc.M113.541318 PMC4031529

[6] Hummler E, Dousse A, Rieder A, Stehle JC, Rubera I, Osterheld MC, et al The channel-activating protease CAP1/Prss8 is required for placental labyrinth maturation. PLoS One. 2013;8(2):e55796 Epub 2013/02/14. doi: 10.1371/journal.pone.0055796 ; PubMed Central PMCID: PMCPMC3565977. 23405214 10.1371/journal.pone.0055796 PMC3565977

[7] List K, Szabo R, Wertz PW, Segre J, Haudenschild CC, Kim SY, et al Loss of proteolytically processed filaggrin caused by epidermal deletion of Matriptase/MT-SP1. J Cell Biol. 2003;163(4):901–10. doi: 10.1083/jcb.200304161 . 14638864 10.1083/jcb.200304161 PMC2173680

[8] List K, Kosa P, Szabo R, Bey AL, Wang CB, Molinolo A, et al Epithelial integrity is maintained by a matriptase-dependent proteolytic pathway. Am J Pathol. 2009;175(4):1453–63. doi: 10.2353/ajpath.2009.090240 . 19717635 10.2353/ajpath.2009.090240 PMC2751542

[9] Szabo R, Peters DE, Kosa P, Camerer E, Bugge TH. Regulation of feto-maternal barrier by matriptase- and PAR-2-mediated signaling is required for placental morphogenesis and mouse embryonic survival. PLoS Genet. 2014;10(7):e1004470 Epub 2014/08/01. doi: 10.1371/journal.pgen.1004470 ; PubMed Central PMCID: PMCPMC4117450. 25078604 10.1371/journal.pgen.1004470 PMC4117450

[10] Planes C, Randrianarison NH, Charles RP, Frateschi S, Cluzeaud F, Vuagniaux G, et al ENaC-mediated alveolar fluid clearance and lung fluid balance depend on the channel-activating protease 1. EMBO molecular medicine. 2010;2(1):26–37. doi: 10.1002/emmm.200900050 ; PubMed Central PMCID: PMC3377187. 20043279 10.1002/emmm.200900050 PMC3377187

[11] Frateschi S, Keppner A, Malsure S, Iwaszkiewicz J, Sergi C, Merillat AM, et al Mutations of the serine protease CAP1/Prss8 lead to reduced embryonic viability, skin defects, and decreased ENaC activity. Am J Pathol. 2012;181(2):605–15. doi: 10.1016/j.ajpath.2012.05.007 . 22705055 10.1016/j.ajpath.2012.05.007

[12] Malsure S, Wang Q, Charles RP, Sergi C, Perrier R, Christensen BM, et al Colon-specific deletion of epithelial sodium channel causes sodium loss and aldosterone resistance. J Am Soc Nephrol. 2014;25(7):1453–64. doi: 10.1681/ASN.2013090936 ; PubMed Central PMCID: PMC4073440. 24480829 10.1681/ASN.2013090936 PMC4073440

[13] Keppner A, Malsure S, Nobile A, Auberson M, Bonny O, Hummler E. Altered Prostasin (CAP1/Prss8) Expression Favors Inflammation and Tissue Remodeling in DSS-induced Colitis. Inflammatory bowel diseases. 2016;22(12):2824–39. doi: 10.1097/MIB.0000000000000940 . 27755216 10.1097/MIB.0000000000000940

[14] Neri I, Virdi A, Tortora G, Baldassari S, Seri M, Patrizi A. Novel p.Glu519Gln missense mutation in ST14 in a patient with ichthyosis, follicular atrophoderma and hypotrichosis and review of the literature. J Dermatol Sci. 2016;81(1):63–6. doi: 10.1016/j.jdermsci.2015.10.012 . 26596219 10.1016/j.jdermsci.2015.10.012

[15] Bauer A, Hiemesch T, Jagannathan V, Neuditschko M, Bachmann I, Rieder S, et al A Nonsense Variant in the ST14 Gene in Akhal-Teke Horses with Naked Foal Syndrome. G3. 2017;7(4):1315–21. doi: 10.1534/g3.117.039511 ; PubMed Central PMCID: PMC5386879. 28235824 10.1534/g3.117.039511 PMC5386879

[16] Avrahami L, Maas S, Pasmanik-Chor M, Rainshtein L, Magal N, Smitt J, et al Autosomal recessive ichthyosis with hypotrichosis syndrome: further delineation of the phenotype. Clin Genet. 2008;74(1):47–53. doi: 10.1111/j.1399-0004.2008.01006.x . 18445049 10.1111/j.1399-0004.2008.01006.x

[17] Basel-Vanagaite L, Attia R, Ishida-Yamamoto A, Rainshtein L, Ben Amitai D, Lurie R, et al Autosomal recessive ichthyosis with hypotrichosis caused by a mutation in ST14, encoding type II transmembrane serine protease matriptase. American Journal of Human Genetics. 2007;80(3):467–77. PubMed PMID: ISI:000244403300007. doi: 10.1086/512487 17273967 10.1086/512487 PMC1821100

[18] Alef T, Torres S, Hausser I, Metze D, Tursen U, Lestringant GG, et al Ichthyosis, follicular atrophoderma, and hypotrichosis caused by mutations in ST14 is associated with impaired profilaggrin processing. J Invest Dermatol. 2009;129(4):862–9. doi: 10.1038/jid.2008.311 . 18843291 10.1038/jid.2008.311

[19] Tanaka H, Nagaike K, Takeda N, Itoh H, Kohama K, Fukushima T, et al Hepatocyte Growth Factor Activator Inhibitor Type 1 (HAI-1) Is Required for Branching Morphogenesis in the Chorioallantoic Placenta. Mol Cell Biol. 2005;25(13):5687–98. doi: 10.1128/MCB.25.13.5687-5698.2005 . 15964823 10.1128/MCB.25.13.5687-5698.2005 PMC1157006

[20] Szabo R, Molinolo A, List K, Bugge TH. Matriptase inhibition by hepatocyte growth factor activator inhibitor-1 is essential for placental development. Oncogene. 2007;26(11):1546–56. doi: 10.1038/sj.onc.1209966 . 16983341 10.1038/sj.onc.1209966

[21] Nagaike K, Kawaguchi M, Takeda N, Fukushima T, Sawaguchi A, Kohama K, et al Defect of hepatocyte growth factor activator inhibitor type 1/serine protease inhibitor, Kunitz type 1 (Hai-1/Spint1) leads to ichthyosis-like condition and abnormal hair development in mice. Am J Pathol. 2008;173(5):1464–75. doi: 10.2353/ajpath.2008.071142 . 18832587 10.2353/ajpath.2008.071142 PMC2570136

[22] Szabo R, Uzzun Sales K, Kosa P, Shylo NA, Godiksen S, Hansen KK, et al Reduced prostasin (CAP1/PRSS8) activity eliminates HAI-1 and HAI-2 deficiency-associated developmental defects by preventing matriptase activation. PLoS Genet. 2012;8(8):e1002937 Epub 2012/09/07. doi: 10.1371/journal.pgen.1002937 ; PubMed Central PMCID: PMCPMC3431340. 22952456 10.1371/journal.pgen.1002937 PMC3431340

[23] Mitchell KJ, Pinson KI, Kelly OG, Brennan J, Zupicich J, Scherz P, et al Functional analysis of secreted and transmembrane proteins critical to mouse development. Nat Genet. 2001;28(3):241–9. doi: 10.1038/90074 . 11431694 10.1038/90074

[24] Szabo R, Hobson JP, Christoph K, Kosa P, List K, Bugge TH. Regulation of cell surface protease matriptase by HAI2 is essential for placental development, neural tube closure and embryonic survival in mice. Development. 2009;136(15):2653–63. doi: 10.1242/dev.038430 . 19592578 10.1242/dev.038430 PMC2709071

[25] Szabo R, Lantsman T, Peters DE, Bugge TH. Delineation of proteolytic and non-proteolytic functions of the membrane-anchored serine protease prostasin. Development. 2016;143(15):2818–28. Epub 2016/07/08. doi: 10.1242/dev.137968 ; PubMed Central PMCID: PMCPMC5004911. 27385010 10.1242/dev.137968 PMC5004911

[26] Friis S, Sales KU, Schafer JM, Vogel LK, Kataoka H, Bugge TH. The protease inhibitor HAI-2, but not HAI-1, regulates matriptase activation and shedding through prostasin. J Biol Chem. 2014;289(32):22319–32. doi: 10.1074/jbc.M114.574400 ; PubMed Central PMCID: PMC4139241. 24962579 10.1074/jbc.M114.574400 PMC4139241

[27] List K, Currie B, Scharschmidt TC, Szabo R, Shireman J, Molinolo A, et al Autosomal ichthyosis with hypotrichosis syndrome displays low matriptase proteolytic activity and is phenocopied in ST14 hypomorphic mice. J Biol Chem. 2007;282(50):36714–23. doi: 10.1074/jbc.M705521200 . 17940283 10.1074/jbc.M705521200

[28] Buzza MS, Netzel-Arnett S, Shea-Donohue T, Zhao A, Lin CY, List K, et al Membrane-anchored serine protease matriptase regulates epithelial barrier formation and permeability in the intestine. Proc Natl Acad Sci U S A. 2010;107(9):4200–5. doi: 10.1073/pnas.0903923107 . 20142489 10.1073/pnas.0903923107 PMC2840089

[29] Friis S, Tadeo D, Le-Gall SM, Jurgensen HJ, Sales KU, Camerer E, et al Matriptase zymogen supports epithelial development, homeostasis and regeneration. BMC Biol. 2017;15(1):46 Epub 2017/06/03. doi: 10.1186/s12915-017-0384-4 ; PubMed Central PMCID: PMCPMC5452369. 28571576 10.1186/s12915-017-0384-4 PMC5452369

[30] Falconer DS, Snell GD. 2 New Hair Mutants, Rough and Frizzy in the House Mouse. J Hered. 1952;43(1):53–7. doi: 10.1093/oxfordjournals.jhered.a106262 PubMed PMID: WOS:A1952UW67400020.

[31] Friis S, Madsen DH, Bugge TH. Distinct Developmental Functions of Prostasin (CAP1/PRSS8) Zymogen and Activated Prostasin. J Biol Chem. 2016;291(6):2577–82. Epub 2016/01/01. doi: 10.1074/jbc.C115.706721 ; PubMed Central PMCID: PMCPMC4742728. 26719335 10.1074/jbc.C115.706721 PMC4742728

[32] Fan B, Brennan J, Grant D, Peale F, Rangell L, Kirchhofer D. Hepatocyte growth factor activator inhibitor-1 (HAI-1) is essential for the integrity of basement membranes in the developing placental labyrinth. Dev Biol. 2007;303(1):222–30. Epub 2006/12/19. doi: 10.1016/j.ydbio.2006.11.005 . 17174946 10.1016/j.ydbio.2006.11.005

[33] Salomon J, Goulet O, Canioni D, Brousse N, Lemale J, Tounian P, et al Genetic characterization of congenital tufting enteropathy: epcam associated phenotype and involvement of SPINT2 in the syndromic form. Hum Genet. 2014;133(3):299–310. doi: 10.1007/s00439-013-1380-6 . 24142340 10.1007/s00439-013-1380-6

[34] Davidson GP, Cutz E, Hamilton JR, Gall DG. Familial enteropathy: a syndrome of protracted diarrhea from birth, failure to thrive, and hypoplastic villus atrophy. Gastroenterology. 1978;75(5):783–90. . 100367

[35] Maetzel D, Denzel S, Mack B, Canis M, Went P, Benk M, et al Nuclear signalling by tumour-associated antigen EpCAM. Nat Cell Biol. 2009;11(2):162–71. doi: 10.1038/ncb1824 . 19136966 10.1038/ncb1824

[36] Maghzal N, Kayali HA, Rohani N, Kajava AV, Fagotto F. EpCAM controls actomyosin contractility and cell adhesion by direct inhibition of PKC. Dev Cell. 2013;27(3):263–77. doi: 10.1016/j.devcel.2013.10.003 . 24183651 10.1016/j.devcel.2013.10.003

[37] Wu CJ, Feng X, Lu M, Morimura S, Udey MC. Matriptase-mediated cleavage of EpCAM destabilizes claudins and dysregulates intestinal epithelial homeostasis. J Clin Invest. 2017;127(2):623–34. Epub 2017/01/18. doi: 10.1172/JCI88428 ; PubMed Central PMCID: PMCPMC5272188. 28094766 10.1172/JCI88428 PMC5272188

[38] Coombs GS, Bergstrom RC, Pellequer JL, Baker SI, Navre M, Smith MM, et al Substrate specificity of prostate-specific antigen (PSA). Chem Biol. 1998;5(9):475–88. Epub 1998/09/30. . 9751643 10.1016/s1074-5521(98)90004-7

[39] Netzel-Arnett S, Currie BM, Szabo R, Lin CY, Chen LM, Chai KX, et al Evidence for a matriptase-prostasin proteolytic cascade regulating terminal epidermal differentiation. J Biol Chem. 2006;281(44):32941–5. doi: 10.1074/jbc.C600208200 . 16980306 10.1074/jbc.C600208200

[40] Friis S, Uzzun Sales K, Godiksen S, Peters DE, Lin CY, Vogel LK, et al A matriptase-prostasin reciprocal zymogen activation complex with unique features: prostasin as a non-enzymatic co-factor for matriptase activation. J Biol Chem. 2013;288(26):19028–39. doi: 10.1074/jbc.M113.469932 ; PubMed Central PMCID: PMC3696676. 23673661 10.1074/jbc.M113.469932 PMC3696676

[41] Chen LM, Zhang X, Chai KX. Regulation of prostasin expression and function in the prostate. Prostate. 2004;59(1):1–12. doi: 10.1002/pros.10346 . 14991861 10.1002/pros.10346

[42] Wakida N, Kitamura K, Tuyen DG, Maekawa A, Miyoshi T, Adachi M, et al Inhibition of prostasin-induced ENaC activities by PN-1 and regulation of PN-1 expression by TGF-beta1 and aldosterone. Kidney Int. 2006;70(8):1432–8. doi: 10.1038/sj.ki.5001787 . 16941024 10.1038/sj.ki.5001787

[43] Kozan PA, McGeough MD, Pena CA, Mueller JL, Barrett KE, Marchelletta RR, et al Mutation of EpCAM leads to intestinal barrier and ion transport dysfunction. Journal of molecular medicine. 2015;93(5):535–45. doi: 10.1007/s00109-014-1239-x ; PubMed Central PMCID: PMC4408367. 25482158 10.1007/s00109-014-1239-x PMC4408367

[44] Mehta S, Nijhuis A, Kumagai T, Lindsay J, Silver A. Defects in the adherens junction complex (E-cadherin/ beta-catenin) in inflammatory bowel disease. Cell Tissue Res. 2015;360(3):749–60. Epub 2014/09/23. doi: 10.1007/s00441-014-1994-6 . 25238996 10.1007/s00441-014-1994-6

[45] Oshima T, Miwa H. Gastrointestinal mucosal barrier function and diseases. J Gastroenterol. 2016;51(8):768–78. Epub 2016/04/07. doi: 10.1007/s00535-016-1207-z . 27048502 10.1007/s00535-016-1207-z

[46] Choi W, Yeruva S, Turner JR. Contributions of intestinal epithelial barriers to health and disease. Exp Cell Res. 2017;358(1):71–7. Epub 2017/03/28. doi: 10.1016/j.yexcr.2017.03.036 . 28342899 10.1016/j.yexcr.2017.03.036 PMC5958612

[47] Szabo R, Hobson JP, List K, Molinolo A, Lin CY, Bugge TH. Potent inhibition and global co-localization implicate the transmembrane Kunitz-type serine protease inhibitor hepatocyte growth factor activator inhibitor-2 in the regulation of epithelial matriptase activity. J Biol Chem. 2008;283(43):29495–504. doi: 10.1074/jbc.M801970200 . 18713750 10.1074/jbc.M801970200 PMC2570866

