# Supplementary figures and images for: Loss of HAI-2 in mice with decreased prostasin activity leads to an early-onset intestinal failure resembling congenital tufting enteropathy
Source: PLoS One. 2018 Apr 4;13(4):e0194660. doi: 10.1371/journal.pone.0194660 (PMC5884512; doi:10.1371/journal.pone.0194660)

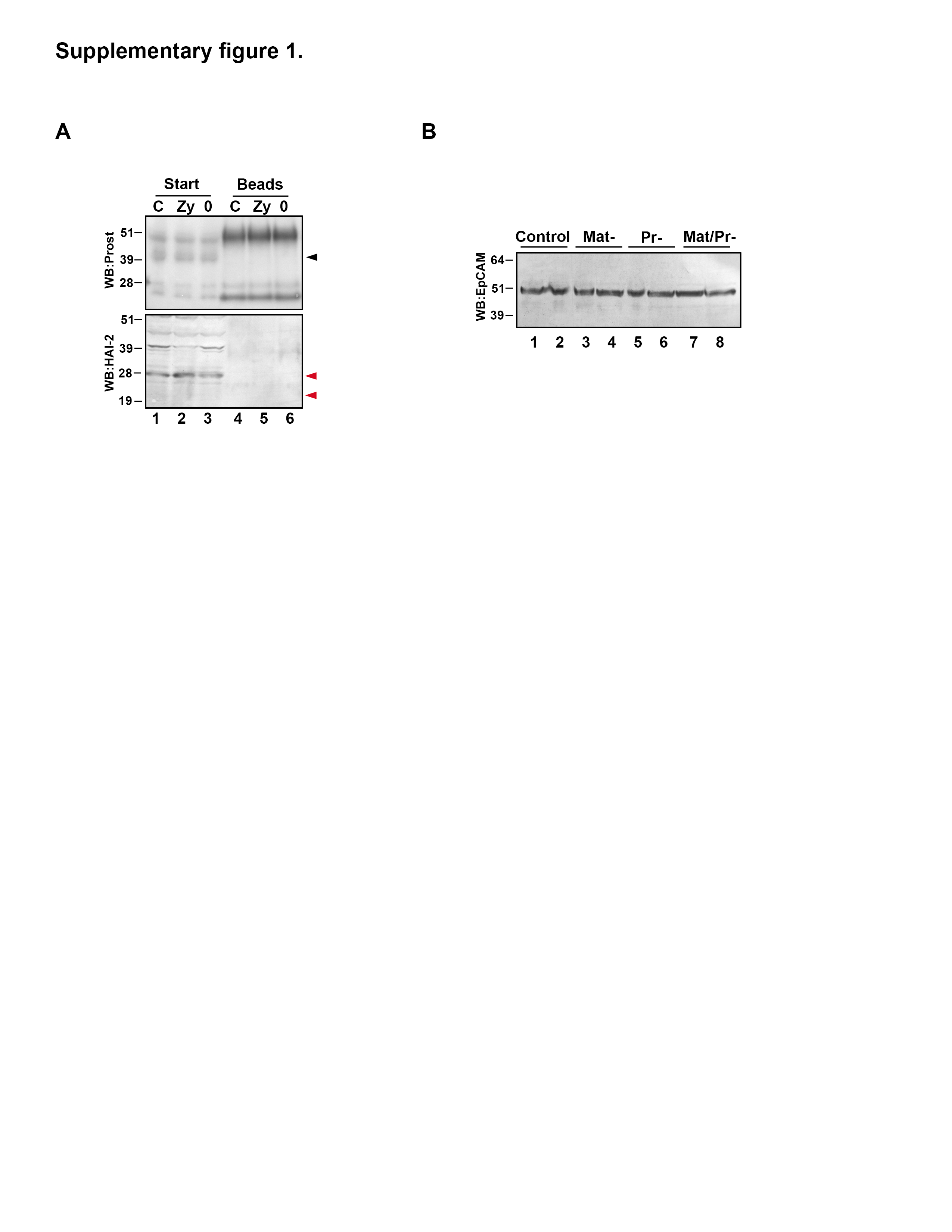

Supplement: S1 Fig — (A). Western blot detection of prostasin (upper panel) and HAI-2 (lower panel) in placental tissue lysates (Start, lanes 1–3) and eluates from GammaBind g Sepharose beads after pre-incubation (Beads, lanes 4–6) from control (Spint2+/+;Prss8+/+, C, lanes 1 and 4), and HAI-2-expressing (Spint2+/+;Prss8R44Q/R44Q (Zy, lanes 2 and 5) or HAI-2-deficient (Spint2-/-; Prss8R44Q/R44Q, 0, lanes 3 and 6) prostasin zymogen-locked embryos used for immunoprecipitation assay shown in Fig 1D. Positions of protein molecular weight markers are shown on the left. Expected size of prostasin and HAI-2 signal is indicated by black arrowhead (top panel) and red arrowheads (bottom panel), respectively. Low concentration and diffuse signal (compare to Fig 1D) prevents clear identification of HAI-2 in the starting material. Neither prostasin nor HAI-2 appear to non-specifically bind sepharose beads. (B). Western blot analysis of EpCAM expression in control (lanes 1 and 2), matriptase-deficient (Villin-Cre+;St14fl/-, lanes 3 and 4), prostasin-deficient (Villin-Cre+;Prss8fl/-, lanes 5 and 6), and matriptase and prostasin double-deficient (Villin-Cre+;St14fl/-; Prss8fl/-, lanes 7 and 8) P2 intestines. No obvious changes in the expression level or proteolytic processing of EpCAM protein have been noticed in any of the tissues. Positions of protein molecular weight markers are shown on the left. (TIF) [file pone.0194660.s001.tif]
