# Supplementary material for: Loss of HAI-2 in mice with decreased prostasin activity leads to an early-onset intestinal failure resembling congenital tufting enteropathy
Source: PLoS One. 2018 Apr 4;13(4):e0194660. doi: 10.1371/journal.pone.0194660 (PMC5884512; doi:10.1371/journal.pone.0194660)
Supplement: S1 Table — (DOCX) [file pone.0194660.s002.docx]

**Supplemental table S1.** Spontaneous phenotypes observed in mice with genetic modification in genes encoding matriptase, prostasin, HAI-1 and HAI-2 and their interactions.

| Mouse model^1^ | Effect on protein biological function | Phenotype | Interactions with other proteins in prostasin/matriptase pathway |
| --- | --- | --- | --- |
| Matriptase |  |  |  |
| *St14^-/-^* | Completely eliminated | Epidermal barrier defect, peri-natal lethality within 48h after birth [[3](#_ENREF_3)] | Perinatal lethality not rescued by elimination of HAI-1 or HAI-2 [[20](#_ENREF_20), [24](#_ENREF_24)] |
| *St14^hypo/hypo^* | Reduced expression | Ichthyosis and hypotrichosis [[27](#_ENREF_27)]  Reduced intestinal barrier function [[28](#_ENREF_28)] |  |
| *St14^R614Q/R614Q^* | Zymogen-locked/ expected reduced [[29](#_ENREF_29)] | Mild skin barrier defect, abnormal hair follicle development [[29](#_ENREF_29)] |  |
| Prostasin |  |  |  |
| *Prss8^-/-^* | Completely eliminated | Epidermal barrier defect, perinatal lethality within 48h after birth [[4](#_ENREF_4)]  Embryonic lethality in some genetic backgrounds [[6](#_ENREF_6)] | Postnatal survival restored by elimination of HAI-1, but not HAI-2 [[25](#_ENREF_25)] |
| *Prss8^fV170D/V170D^* (frizzy) | Expected reduced[[11](#_ENREF_11), [22](#_ENREF_22)] | Wavy coat, curly vibrissae [[30](#_ENREF_30)]. |  |
| *Prss8^S238A/S238A^* | Proteolytically-inactive/expected reduced | Mild skin barrier defect. Kinked, short whiskers, sparse pelage hairs [[5](#_ENREF_5)] |  |
| *Prss8^R44Q/R44Q^* | Zymogen-locked/ expected reduced [[5](#_ENREF_5), [31](#_ENREF_31)] | Mild skin barrier defect. Kinked, short whiskers, sparse pelage hairs [[31](#_ENREF_31)] |  |
| HAI-1 |  |  |  |
| *Spint1^-/-^* | Completely eliminated | Embryonic lethality due to placental defect [[19](#_ENREF_19), [32](#_ENREF_32)] | Placental defects and embryonic lethality prevented by elimination of matriptase (*St14^-/-^*) or prostasin (*Prss8^fr/fr^* or *Prss8^-/-^*, but not *Prss8^S238A/S238A^*) activity [[20](#_ENREF_20), [22](#_ENREF_22), [25](#_ENREF_25)] |
|  |  | Ichthyosis, abnormal hair development, post-natal lethality by day16 [[21](#_ENREF_21)] | Post-natal skin and hair development and survival all restored by reduced activity of matriptase in *St14^hypo/hypo^* background [[24](#_ENREF_24)]  Postnatal survival largely restored in the absence of prostasin (*Prss8^-/-^*), skin- and hair-related phenotypes still present [[25](#_ENREF_25)] |
| HAI-2 |  |  |  |
| *Spint2^-/-^* | Completely eliminated | Early embryonic lethality [[23](#_ENREF_23)] | Prevented by expressing less than two wildtype alleles of matriptase (*St14^+/-^* or *St14^-/-^*) or by reducing prostasin activity (*Prss8^-/-^*, *Prss8^fr/fr^*, *Prss8^R44Q/R44Q^*, but not *Prss8^S238A/S238A^*) ([[22](#_ENREF_22), [24](#_ENREF_24), [25](#_ENREF_25)], this study) |
|  |  | Placental defects, neural tube defects, mid-gestation embryonic lethality [[24](#_ENREF_24)] | Prevented by a complete loss of matriptase (*St14^-/-^*) or by reducing prostasin activity (*Prss8^-/-^*, *Prss8^fr/fr^*, *Prss8^R44Q/R44Q^*, but not *Prss8^S238A/S238A^*) ([[22](#_ENREF_22), [24](#_ENREF_24), [25](#_ENREF_25)], this study) |

^1^ Only mouse models with partial or complete loss-of-function are listed.
