# Supplementary material for: Loss of HAI-2 in mice with decreased prostasin activity leads to an early-onset intestinal failure resembling congenital tufting enteropathy
Source: PLoS One. 2018 Apr 4;13(4):e0194660. doi: 10.1371/journal.pone.0194660 (PMC5884512; doi:10.1371/journal.pone.0194660)
Supplement: S2 Table — (DOCX) [file pone.0194660.s003.docx]

**Table S2.** List of antibodies used in the study

| **Immunohistochemistry**  **Primary** | | |  |  |
| --- | --- | --- | --- | --- |
| **Antigen** | **Manufacturer** | | **Cat. No.** | **Concentration** |
| Ki67 | Novocastra, Leica Biosystems, Newcastle, UK | | NCL-Ki67p | 1:500 |
| Cleaved caspase-3 | Cell Signaling Technology, Danvers, MA | | 9664 | 1:100 |
| **Secondary** | | |  |  |
| Anti-rabbit | Vector Laboratories, Burlingame, CA | | BA-1000 | 2.5 ug/ml |
|  | |  |  |  |
|  | |  |  |  |
| **Western Blot**  **Primary** | | |  |  |
| HAI-2 | R&D Systems, Minneapolis, MN | | AF1107 | 1 ug/ml |
| Prostasin^1^ | BD Transduction Laboratories | | 612173 | 1 ug/ml |
| Prostasin^2^ | R&D Systems, Minneapolis, MN | | AF4599 | 1 ug/ml |
| EpCAM | R&D Systems, Minneapolis, MN | | AF960 | 2 ug/ml |
| E-cadherin | R&D Systems, Minneapolis, MN | | AF648 | 2 ug/ml |
| Claudin-1 | Abcam, Cambridge, MA | | ab15098 | 2 ug/ml |
| Claudin-2 | Abcam, Cambridge, MA | | ab53032 | 2 ug/ml |
| Claudin-4 | Invitrogen, Carlsbad, CA | | 36-4800 | 2 ug/ml |
| Claudin-7 | Invitrogen, Carlsbad, CA | | 34-9100 | 2 ug/ml |
| Occludin | Invitrogen, Carlsbad, CA | | 71-1500 | 2 ug/ml |
| GAPDH | Cell Signaling Technology, Danvers, MA | | 2118 | 1 ug/ml |
|  |  | |  |  |
| **Secondary** | |  |  |  |
| Anti-sheep | Sigma, St.Louis, MO | | A5187 | 1 ug/ml |
| Anti-mouse | Dako Cytomation, Carpinteria, CA | | D0486 | 1 ug/ml |
| Anti-rabbit | Dako Cytomation, Carpinteria, CA | | D0487 | 1 ug/ml |
| Anti-goat | Sigma, St.Louis, MO | | A4187 | 1 ug/ml |
|  |  | |  |  |
| **Immunoprecipitation** | | | | |
| HAI-2 | R&D Systems, Minneapolis, MN | | AF1107 | 5 ug/ml |
| Prostasin | R&D Systems, Minneapolis, MN | | AF2968 | 5 ug/ml |
|  |  | |  |  |

^1^ used for Western blot detection of mouse prostasin after immunoprecipitation (Figure 1D)

^2^ used for Western blot detection of free and complexed human prostasin (Figure 1A-1C)
