# Supplementary material for: Loss of HAI-2 in mice with decreased prostasin activity leads to an early-onset intestinal failure resembling congenital tufting enteropathy
Source: PLoS One. 2018 Apr 4;13(4):e0194660. doi: 10.1371/journal.pone.0194660 (PMC5884512; doi:10.1371/journal.pone.0194660)
Supplement: S3 Table — (DOCX) [file pone.0194660.s004.docx]

**Table S3.** Sequences of PCR primers used for reverse transcription quantitative PCR

| **Gene** | **Primer sequences** | **Reference** |
| --- | --- | --- |
| **EpCAM**  **E-cadherin**  **Claudin-1**  **Claudin-2**  **Claudin-4**  **Claudin-7**  **Occludin**  **S15** | 5’-aagcagaaatgactcacagc-3’  5’-atcgagatgtgaacgcctc-3’  5’-tcatggatcagaagatcacg-3’  5’-ggcagaactgcatgtttcg-3’  5’-cgcaatctttgtgtccacc-3’  5’-agcaggaaagtaggacacc-3’  5’-ttagccctgaccgagaaaga-3’  5’-aaaggacctctctggtgctg-3’  5’-agagcacaggtcagatgc-3’  5’-atagggttgtagaagtcgc-3’  5’-gtctgctctggtccttctgg-3’  5’-aaagcacaccccatgtcttc-3’  5’-actaccttgggtgctgtgct-3’  5’-aaattgggctggatgtaat-3’  5’-ttccgcaagttcacctacc-3’  5’-cgggccggccatgctttacg-3’ | this study  this study  this study  [1]  this study  [1]  [1]  RETROscript Kit, Applied Biosystems |

**Reference:**

# [1] van den Bossche et al. (2012). Claudin-1, Claudin-2 and Claudin-11 Genes Differentially Associate with Distinct Types of Anti-inflammatory Macrophages *In vitro* and with Parasite- and Tumour-elicited Macrophages *In vivo*. Scandinavian Journal of Immunology 75; 588-598.
